# Radiographic and Histomorphometric Evaluation of Sinus Floor Augmentation Using Biomimetic Octacalcium Phosphate Alloplasts: A Prospective Pilot Study

**DOI:** 10.3390/ma15124061

**Published:** 2022-06-07

**Authors:** Seok-Jun Kim, Joo-Seong Kim, Woong Kim, Suk-Young Kim, Won-Pyo Lee

**Affiliations:** 1 Department of Biomedical Science, College of Natural Sciences, Chosun University, Gwangju 61452, Korea; heaven1472@chosun.ac.kr (S.-J.K.); kw@chosun.ac.kr (W.K.); 2 Department of Biomedical Engineering, Yeungnam University, Daegu 42415, Korea; joorpediem@gmail.com; 3 School of Materials Science and Engineering, Yeungnam University, Gyeongsan 38541, Korea; sykim@ynu.ac.kr; 4 Department of Periodontology, School of Dentistry, Chosun University, Gwangju 61452, Korea

**Keywords:** bone regeneration, clinical study, octacalcium phosphate, sinus floor augmentation

## Abstract

This prospective single-arm clinical study aimed to radiographically and histomorphometrically evaluate the efficacy of the lateral approach for sinus floor elevation (LSFE) using biomimetic octacalcium phosphate (OCP) synthetic bone graft (Bontree^®^). LSFE using Bontree^®^ was performed on 10 patients (15 implant placement sites) willing to undergo implant surgery, followed by implant placements after 6 months of the healing period. The vertical bone height (VBH) and Hounsfield unit (HU) values at each implant placement site were evaluated radiographically using cone-beam computed tomography at baseline immediately after surgery (T1) and 6 months after surgery (T2). A histomorphometric evaluation of the bone core biopsy specimen was also performed. The mean VBH and HU changes at all sites included a decrease by 0.91 mm and a statistically significant increase by 431.86, respectively, from T1 to T2. The mean ratio of the newly formed bone (23.34% ± 10.63%) was greater than that of the residual bone graft (19.09% ± 8.74%), indicating that Bontree^®^ is effective for new bone formation. This pilot study suggests that Bontree^®^ is a promising bone substitute for LSFE.

## 1. Introduction

After maxillary molar loss, the absorption of the horizontal and vertical alveolar ridge and the pneumatization of the maxillary sinus appear, serving as a limitation to implant placement [1]. Maxillary sinus floor elevation has been accepted as one of several pre-prosthetic surgical procedures to circumvent the limitations of standard implant placement procedures due to the insufficient residual alveolar height in the maxillary posterior region. The lateral approach for sinus floor elevation (LSFE) was first introduced by Tatum [2] in 1977 and was first announced by Boyne and James [3] in 1980. It has been considerably developed with the advancement of surgical techniques and materials for approximately 40 years. The paradigm for the use of bone graft materials has changed from a previous study claiming the employment of only autogenous bone to the concept that more significant implant survival rates can be obtained when bone replacement grafts are used according to a systematic review of the literature [4]. Allografts, xenografts, and synthetic grafts are considered attractive alternatives that could be employed in order to overcome the shortcomings of autologous bone grafts [4]. Our previous study has shown histologically comparable new bone formation and sufficient sinus floor augmentation using allografts rather than xenografts [5]. Synthetic bone graft materials, mainly made of ceramic, tricalcium phosphate, and hydroxyapatite (HA), have the advantages of mass production, the control of properties, and no risk of cross-infection. 

Recently, octacalcium phosphate (OCP, Ca_8_H_2_[PO_4_]_6_ 5H_2_O) was developed as a new synthetic bone graft material [6]. OCP, a direct precursor of biological apatite, has proven effective for new bone formation due to its rapid biodegradability and high osteogenic capacity [7]. Despite these advantages, developing a commercial bone substitute is difficult because of the sintering process at a high temperature, which is generally required during the manufacturing process of synthetic substitute materials, thereby changing the original crystal structure of OCP, which loses its intrinsic properties [8]. However, the recent developments in mass production technology at room temperature, which does not require a sintering process, have opened up the possibility of clinical applications with improved physical properties and an inherently high osteogenic capacity of OCP. Our previous studies have demonstrated the efficacy and safety of bone regeneration using this commercialized biomimetic OCP synthetic bone substitute material (Bontree^®^; HudensBio Co., Gwangju, Korea) in preclinical and clinical studies [9,10]. 

Based on our previous studies, this pilot study aimed to evaluate the radiographic and histomorphometric efficacy of maxillary sinus floor augmentation using the commercial OCP synthetic bone substitute material Bontree^®^.

## 2. Materials and Methods

### 2.1. Study Design 

This was a prospective, single-arm, monocenter clinical study. The research protocol was approved by the Institutional Review Board (IRB) of the Chosun University Dental Hospital (CUDHIRB-2101-006). All of the registered participants provided written informed consent, and the study was conducted in accordance with the Helsinki Declaration and the Good Clinical Practice guidelines.

### 2.2. Study Population

From January 2021 to June 2021, this clinical trial recruited 10 participants who needed implant surgery after LSFE at the Department of Periodontology, Chosun University Dental Hospital. The inclusion criteria were: (a) 18 years of age or older; (b) less than 5 mm of residual bone height (RBH) from the maxillary sinus floor, requiring staged implant placement; and (c) systemic health or well-controlled conditions for LSFE and implant surgery. The exclusion criteria were: (a) uncontrolled periodontal disease; (b) bone metabolic disease; (c) heavy smoking (>10 cigarettes/day); and (d) contraindications for LSFE and implant surgery due to other systemic disorders.

### 2.3. Surgical Procedure 

A skilled periodontologist (W-P.L.) performed all of the surgical procedures. 

**Figure 1:**
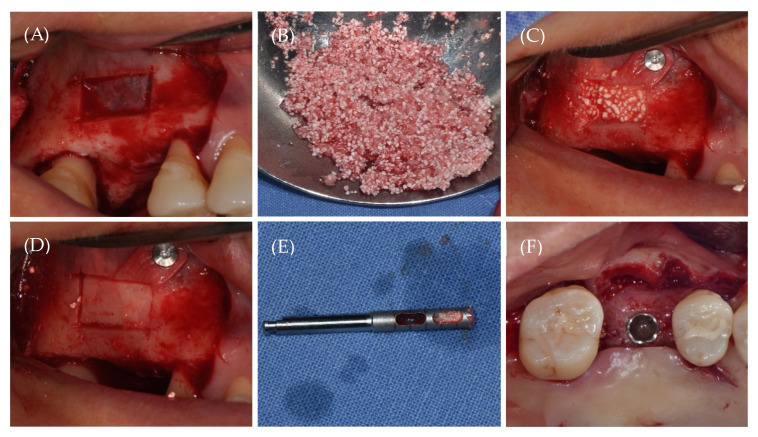
The surgical procedure. (**A**) A piezoelectric bony window osteotomy, (**B**) the mixture of biomimetic Octacalcium Phosphate (OCP) alloplasts and whole blood, (**C**) the sinus graft, (**D**) the repositioned bony lid, (**E**) the core biopsy harvested using a trephine bur after 6 months of healing, (**F**) the implant fixture placement.

Under local anesthesia, the full-thickness flap was elevated to expose the lateral wall of the maxillary sinus after a mid-crestal incision and vertical releasing incision using a #15c blade. A bony window osteotomy was performed using a piezoelectric device (Figure 1A). After the sinus membrane elevation, a mixture of biomimetic OCP-based Bontree^®^ bone substitute material and whole blood was grafted into the maxillary sinus (Figure 1B,C). The bony lid was then repositioned (Figure 1D), and primary closure was carried out. 

After 6 months of healing after LSFE, a core biopsy (diameter 2.0 mm, length 8.0 mm) was harvested using a trephine bur before drilling the implant placement site (Figure 1E). Subsequently, the internal-type implant for the site was placed (Figure 1F), and the primary suture was performed.

### 2.4. Results Analysis

#### 2.4.1. Clinical Evaluation 

Postoperative complications, such as infection, pain, edema, and flap dehiscence, were evaluated at 1 week, 2 weeks, 6 weeks, 3 months, and 6 months after LSFE. The implant stability quotient (ISQ) for all implants was measured at the first-stage surgery.

#### 2.4.2. Radiological Evaluation 

**Figure 2:**
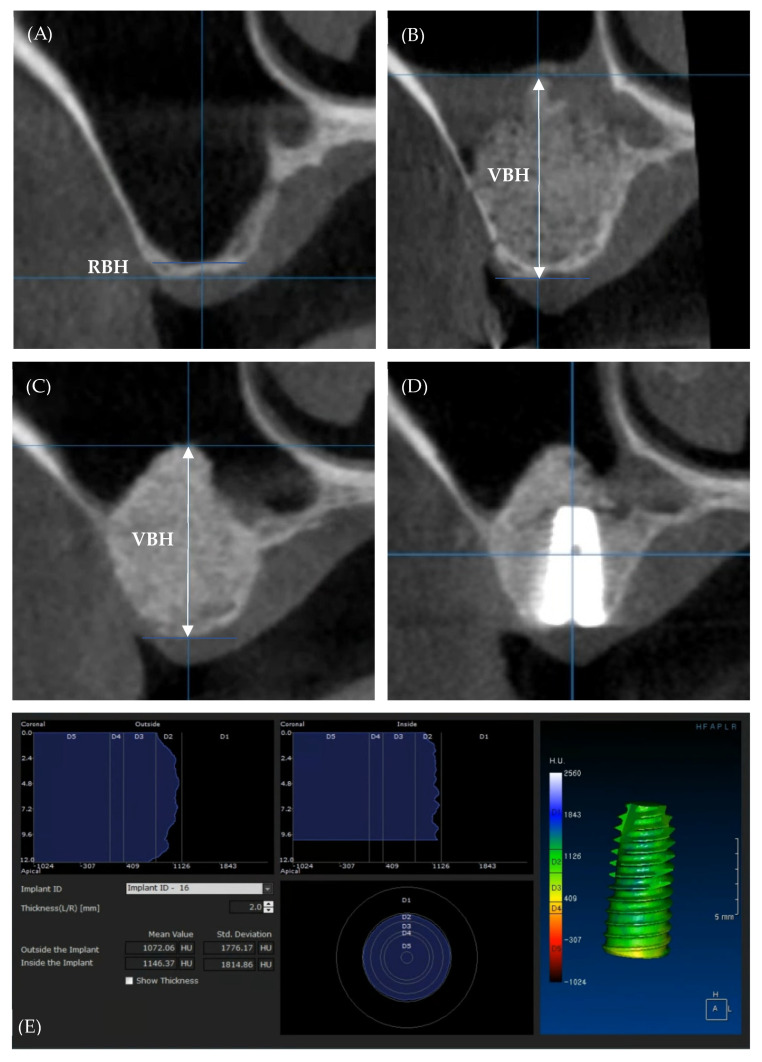
Cone−beam computed tomography images according to the implant placement site. (**A**) Preoperatively (T0), (**B**) immediately after the surgery (T1), (**C**) 6 months postoperatively (T2), (**D**) after implant placement, (**E**) Hounsfielid unit (HU) value measurement. RBH, residual bone height; VBH, maximum vertical bone height.

For radiographic evaluation, cone-beam computed tomography (CBCT) was conducted for sinus floor augmentation under the same imaging conditions (field of view (FOV) height, 5.6 cm; FOV diameter, 10 cm; voxel size, 0.2 mm; beam currency, 8.0 mA; acceleration voltage, 90 kV) preoperatively (T0), as well as immediately after the surgery (T1) and 6 months postoperatively (T2, just before the first-stage surgery). The residual bone height (RBH) and maximum vertical bone height (VBH) were measured using software (OnDemand3D^TM^; Cybermed, Seoul, Korea) according to the implant placement sites. The Hounsfield unit (HU) values were also measured based on the automatic tools of the same software according to Tallarico et al. [11] (Figure 2). All of the measurements were performed thrice by an independent investigator (W.K.) not involved in the surgery, and the average value was used.

#### 2.4.3. Histomorphometric Evaluation 

The harvested specimen was fixed using 4% buffered saline with paraformaldehyde for at least 2 days and then demineralized for at least 2 weeks. The specimen was then processed into paraffin blocks, and a microtome was used to make 5 µm thick microsectioning. Next, trichrome staining was performed, and the images of the samples were obtained using a camera connected to a light microscope. For the histomorphometric analysis, the proportions of newly formed bone (NB), residual graft material (RG), and connective tissue (CT) were measured using i-Solution software (IMT i-Solution Inc., Burnaby, BC, Canada). These measurements were performed thrice by an independent examiner (J-S.K.) who was not involved in the surgery, and the average value was used. 

#### 2.4.4. Statistical Analyses

The normality of the data distribution was evaluated using the Shapiro–Wilk test. Confirming that the normal distribution was not followed, all of the results were analyzed using nonparametric tests, which were the Wilcoxon signed-rank test and the Friedman test. The statistical significance level was set at *p* < 0.05. Under the review of the methodology by an independent statistician, all of the statistical analyses were carried out using SPSS version 25.0 (SPSS Inc., Chicago, IL, USA). 

## 3. Results

### 3.1. Patient Characteristics 

Based on the inclusion and exclusion criteria, 10 patients with an overall mean age of 59.1 years (range, 36–74 years) were enrolled in this study. A total of 15 sites of implant placement in all of the patients were clinically and radiologically evaluated (Table 1).

### 3.2. The Clinical Evaluation Results 

The results are presented in Table 2. The average ISQ value was 65.90 ± 3.37, and the initial fixation of all the implants (n = 15) was stable (ISQ > 60). No severe complications or unusual events, excluding general mild pain or edema postoperatively, were noted during the surgery and follow-up. 

### 3.3. The Radiological Evaluation Results 

**Figure 3:**
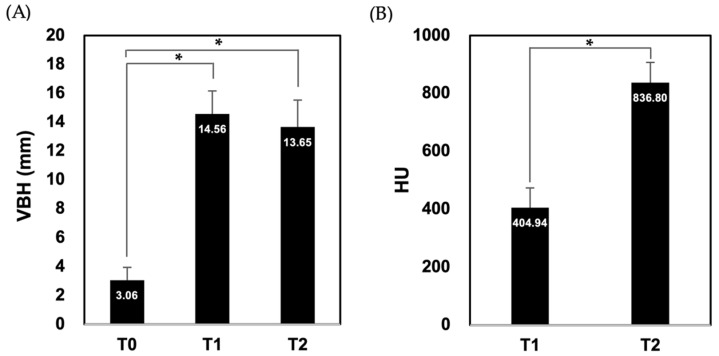
Radiological evaluation at the implant placement site. (**A**) Residual bone height (T0) and maximum vertical bone height (VBH) immediately (T1) and 6 months (T2) after surgery, (**B**) the Hounsfield Unit (HU). The statistical significance was set at * *p* < 0.05 by the Wilcoxon signed-rank test and the Friedman test.

The results are presented in Figure 3. The average values of RBH and VBH were 3.06 ± 0.89 mm (T0), 14.56 ± 1.60 mm (T1), and 13.65 ± 1.88 mm (T2), indicating statistically significant differences among the times. The HU values at T1 and T2 were 404.94 ± 67.41 mm and 836.80 ± 70.31 mm, respectively, indicating a statistically significant difference between the times.

### 3.4. The Histomorphometric Evaluation Results 

**Figure 4:**
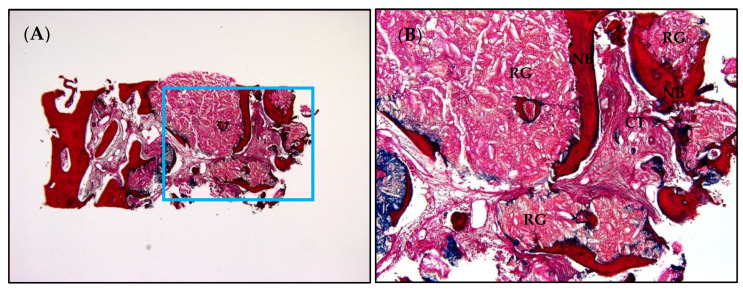
Histologic view and histomorphometric analysis. (**A**) Residual graft material (RG) is circumscribed by the newly formed bone (NB) (trichrome staining; magnification, ×40), (**B**) NB showing no inflammatory tissue (trichrome staining; magnification, ×100). CT, connective tissue.

Eight of the fifteen specimens from the 10 patients were discarded because there was not sufficient bone graft material for evaluation in the harvested specimens. Therefore, seven samples from seven patients were processed for the histomorphometric analysis. NB, RG, and CT without inflammatory tissue were observed in the core biopsy samples (Figure 4). The results of the histomorphometric analysis are presented in Table 3. The mean proportions of NB, RG, and CT were 23.34% ± 10.63%, 19.09% ± 8.74%, and 57.57% ± 14.47%, respectively.

## 4. Discussion

In this clinical study, the radiographic and histomorphometric results of sinus floor augmentation using a biomimetic OCP synthetic bone-substitute material were evaluated. Currently, only a few commercially available OCP-based products, such as Ti-Oss^®^ (Chiyewon, Guri, Korea), exist. These are demineralized bovine bone materials (DBBM) coated with OCP or Bonarc^TM^ (Toyobo Co. Ltd., Osaka, Japan), which is a mixture of OCP and collagen. However, unlike these products, which are not fully composed of Ca- and P-containing apatites, the recently introduced OCP bone graft Bontree^®^ is fully composed of apatite compounds with 80 wt.% OCP and 20 wt.% HA [9]. Therefore, to our knowledge, this is the first clinical study on maxillary sinus grafts using a pure OCP-based synthetic bone substitute.

The ideal bone substitute material for sinus floor augmentation should stabilize the space for new bone formation and osseointegration and should be maintained until the maturation of the regenerated bone after prosthetic loading [12]. DBBM is commonly used as an alternative material for the sinus grafting of autogenous bones [13,14]. DBBM can serve as a scaffold for new bone ingrowth and maintain the space beneath the elevated sinus membrane because it has excellent osteoconductivity and is slowly biodegraded [15,16]. DBBM has been reported to maintain augmented bone height, preventing pneumatization, for more than 3 years [17]. Similarly, our recent study showed almost no change in VBH, with a decrease of only 0.14 mm after 6 months of sinus grafting using DBBM. On the other hand, when 100% cancellous freeze-dried bone allograft (FDBA) and mixed FDBA (cortical FDBA 50%: cancellous FDBA 50%) were used, 2.16 mm and 1.30 mm decreases in VBH were observed 6 months after surgery, respectively [5]. In the case of synthetic bone substitute materials, biphasic calcium phosphate (BCP) bioceramics, which consist of HA and beta-tricalcium phosphate, are currently the most widely used [18]. BCP in the maxillary sinus maintains its volume well and is surrounded by the lamellar bone, confirming dimensional stability and successful bone formation [19]. Mordenfeld et al. reported that the decreases in VBH after sinus grafting were 0.7 mm and 1.0 mm after 2 years and 6 years of healing, respectively. Meanwhile, Miura et al. reported that Bonarc^TM^, an OCP and collagen mixture, is a synthetic bone graft material and showed a relatively large tendency of VDH reduction—1.5 mm from 3 months to 12 months of healing after sinus floor augmentation [20]. In contrast, in this study, the decrease in VDH was 0.91 mm after healing for 6 months following sinus grafting, despite using the OCP-based bone graft material Bontree^®^. This difference is because Bonarc^TM^ is a synthetic bone graft material in which OCP is mixed with collagen, which is generally highly absorbable, while Bontree^®^ is composed of apatite compounds with 80 wt.% OCP and 20 wt.% HA. In the case of Bontree^®^, the HU value rose from 404.94 immediately after surgery to 836.80 after 6 months, suggesting progression in the maturation of bone quality and the appropriate quality to maintain the implant. In the case of Bonarc^TM^, it also increased from 231.6 mm at 3 months post-surgery to 294.4 mm at 6 months post-surgery, showing a similar trend.

Bone density related to bone-implant contact must be sufficient to achieve higher implant survival rates after sinus floor elevation. Therefore, the histological evaluation of the NB amount after maxillary sinus grafting using bone substitute materials is critical [21]. The compositions of NB and RG may vary according to the study design and observation period. Danesh-Sani et al. has reported that the NB ratios can range from 19% to 44% based on a healing period of 9–13.5 months and of 4.5–9 months after sinus grafting using various bone graft materials in a systematic review [22]. Analyzed bone cores showing more NB than RG indicate the superior new bone formation capacity of the bone substitute material [23]. In this study, the mean ratios of NB and RG were 23.34% ± 10.63% and 19.09% ± 8.74%, respectively, indicating that Bontree^®^ is effective for new bone formation due to the high osteogenic capacity of OCP.

The results of this study should be interpreted carefully because the number of subjects (n = 10) is relatively small and the follow-up time (6 months) is short. In addition, this was a single-arm pilot study with no control group. In this study, the unit of HU should also be interpreted with caution. In fact, CT could be calibrated using the density values of the air (−1000 HU) and pure water (0 HU) as a reference; otherwise, CBCT does not consent to be calibrated, and the values, which are based on the difference in the gray scale, are already preset by the manufacturer. Finally, since the VBH was measured only two-dimensionally, further research is needed in the future to evaluate three-dimensional changes.

## 5. Conclusions

Our study concluded that, when sinus grafting was performed using a biomimetic OCP-based synthetic bone substitute material, the VBH and HU values could be sufficiently increased during the 6-month healing period. In addition, the histomorphometric analysis indicates the potential for new bone formation due to the inherently high osteogenic capacity of OCP.

## Figures and Tables

**Table 1:** Patient characteristics.

Case	Gender	Age (In Years)	Sinus	Implant Placement Site	Implant Type	Fixture Diameter/Length (mm/mm)
1	F	50	Rt	16	Sinheung (Luna)	4.5 × 10
2	F	74	Rt	16	Sinheung (Luna)	4.5 × 10
3	F	71	Rt	16	Megagen (Anyone)	5.0 × 8.5
4	F	69	Rt	17	Megagen (Anyone)	4.5 × 8.5
5	F	55	Lt	26	Sinheung (Luna)	4.5 × 10
6	M	36	Lt	25	Sinheung (Luna)	4.0 × 10
7	F	65	Lt	26	Megagen (Anyone)	5.0 × 10
				27	Megagen (Anyone)	5.0 ×10
8	M	58	Lt	26	Dentium (Superline)	5.0 × 10
				27	Dentium (Superline)	5.0 × 8.5
9	M	49	Rt	15	Dentium (Superline)	4.5 × 10
				16	Dentium (Superline)	4.5 × 10
				17	Dentium (Superline)	4.5 × 10
10	M	64	Rt	16	Dentium (Superline)	5.0 × 12
				17	Dentium (Superline)	5.0 × 12

Rt, right side; Lt, left side.

**Table 2:** A clinical evaluation of the implant stability quotient (ISQ) value.

Case	1	2	3	4	5	6	7		8		9			10		Mean ± SD
Site	16	16	16	17	26	25	26	27	26	27	15	16	17	16	17	
ISQ	66.0	69.0	65.0	63.5	62.0	70.5	68.0	64.0	71.0	65.0	70.0	68.0	64.0	62.5	60.0	65.90 ± 3.37

Site, implant placement site; SD, standard deviation.

**Table 3:** Histomorphometric evaluation of each specimen.

Case	1	2	3	5	6	8	9	Mean ± SD
Site	16	16	16	26	25	26	16	
NB	30.90	13.70	39.20	13.00	23.10	12.70	30.80	23.34 ± 10.63
RG	31.00	30.30	13.60	7.40	15.90	15.50	19.90	19.09 ± 8.74
CT	38.10	56.00	47.20	79.60	61.00	71.80	49.30	57.57 ± 14.47

NB, newly formed bone; RG, residual graft material; CT, connective tissue; Site, implant placement site; SD, standard deviation. Data are represented in percentage values.

## Data Availability

The datasets generated or analyzed during the current study are available from the corresponding author on reasonable request.
